# A Rare Case of Mediastinal Mass: Thymoma and Thymic Tumor

**DOI:** 10.7759/cureus.40956

**Published:** 2023-06-25

**Authors:** Sofia Moura de Azevedo, Patrícia Baptista, Rita C Pichel, Rita R Dias, Manuela Bertão

**Affiliations:** 1 Internal Medicine, Centro Hospitalar Universitário de Santo António, Porto, PRT; 2 Oncology, Centro Hospitalar Universitário de Santo António, Porto, PRT

**Keywords:** radiotherapy, tumor boards, thoracic surgery, mediastinal neoplasms, thymic carcinomas, thymomas

## Abstract

Thymomas and thymic carcinomas are rare mediastinal neoplasms arising from thymic epithelial cells, and the presence of synchronous or metachronous primary thymic neoplasms in a single patient is an extremely rare event. Thymoma patients appear to have an inherent predisposition toward developing additional neoplasms. This additionally presents a diagnostic challenge, revealing the importance of multidisciplinary expertise to the management of these patients. This is a case report of a patient with a thymoma and thymic carcinoma, submitted to surgical resection and postoperative radiotherapy.

## Introduction

Thymic epithelial cell-derived neoplasms, namely thymomas and thymic carcinomas, are infrequent tumors found in the mediastinum. Thymomas have non-neoplastic lymphocytes in a variable number. When we find epithelial tumor cells with overt cytologic atypia, invasiveness, and lack of thymus-like features of thymomas, we have a thymic carcinoma [1-4]. Thymoma represents only 0.2-1.5% of all malignancies, with no gender differences [2]. Surgery is curative in most cases, some requiring adjuvant radiotherapy. Thymic carcinomas present a worse prognosis compared with thymomas, with a median time to death of under three years [5-7].

## Case presentation

**Figure 1:**
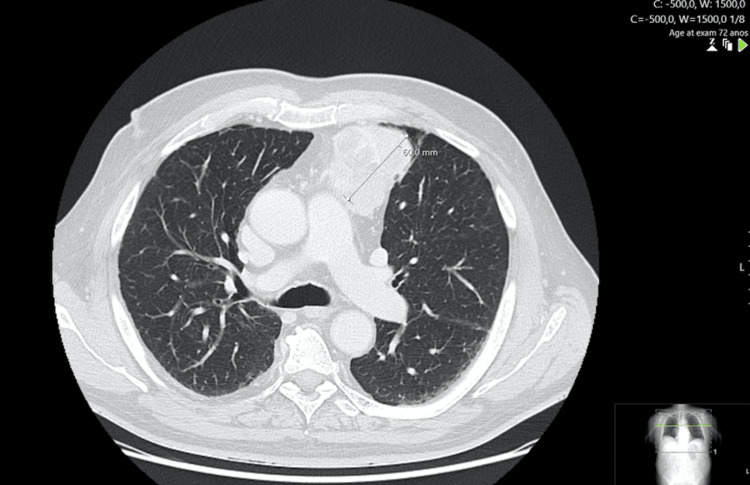
Thoracic computerized tomography scan, axial view, evidencing the mass in the anterior mediastinum (transverse axis measured).

A 73-year-old Caucasian male, who was submitted to cholecystectomy because of acute cholangitis, had an incidental finding of a mediastinal mass in the imagiologic evaluation. The patient had no other relevant past medical history. Computerized tomography (CT) scan revealed the presence of a heterogeneous lobulated mass in the anterior mediastinum, with some more hypodense areas, with approximately 68 mm of the transverse axis, 74 mm of the anteroposterior axis, and approximately 63 mm of the longitudinal axis. It presented regular and well-defined contours, without calcifications or fat (Figure 1).

**Figure 2:**
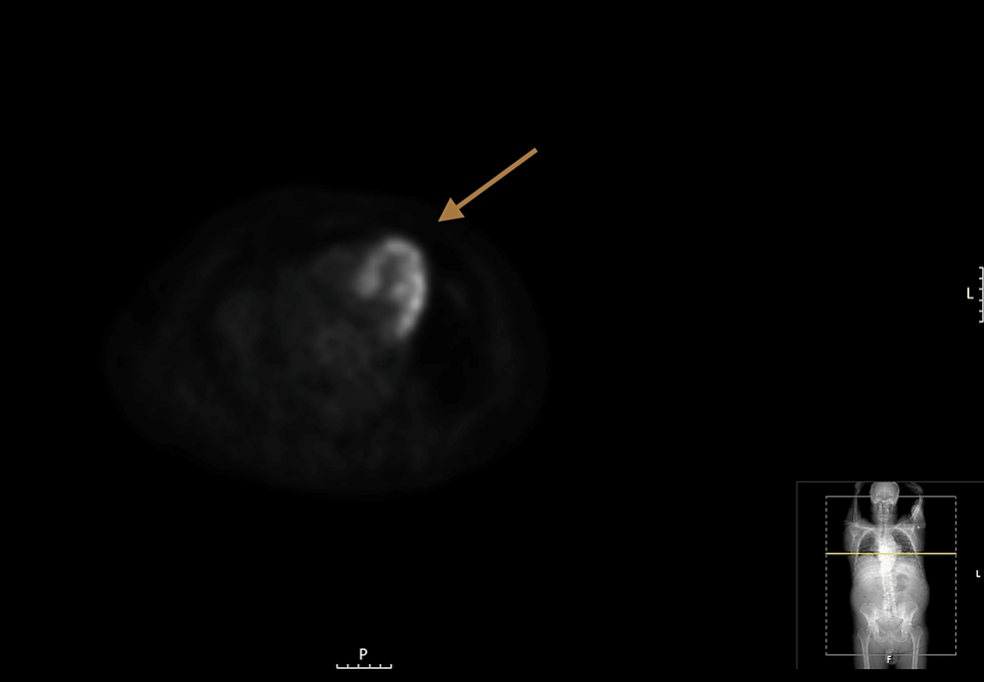
PET-CT scan, axial view, showing the anterior mediastinal mass with moderate-to-intense avidity for FDG. PET-CT, positron emission tomography computerized tomography; FDG, fluorodeoxyglucose

Blood workup did not show cytopenias nor elevated inflammation parameters, and serologic tumor markers were negative. Positron emission tomography CT (PET-CT) scan showed an anterior mediastinal mass with moderate-to-intense avidity for fluorodeoxyglucose (FDG) (Figure 2), and no other site of increased FDG avidity.

**Figure 3:**
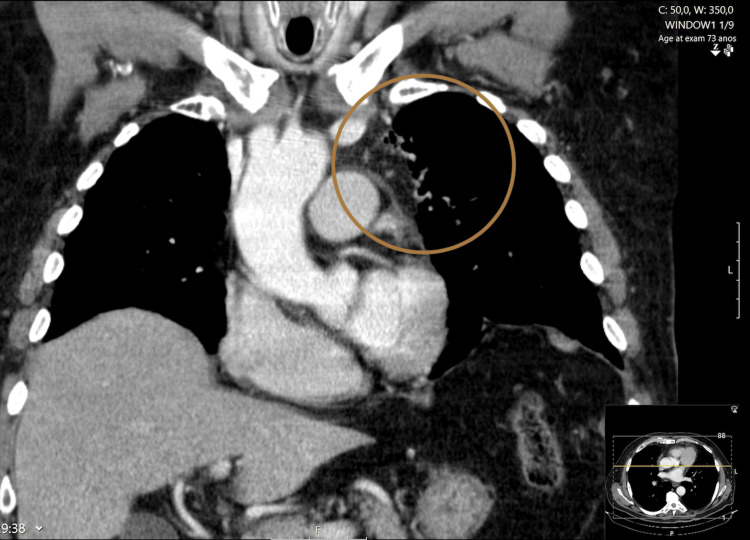
Thoracic computerized tomography scan, coronal view, after surgical removal of the mass.

The case was presented and discussed in a thoracic tumor board and decided to proceed to surgical resection of the mass for diagnostic and therapeutic purposes (Figure 3).

Tumorectomy via sternotomy was performed and the mass was described as multilobulated with white hemorrhagic areas of necrosis. The anatomopathological evaluation found thymic neoplasm patterns and focus of epidermoid carcinoma, with extension to the thymic fat. In the immunohistochemical study, CK5/6, p63, CK19, and EMA (focal) expression were observed in epidermoid carcinoma. Due to the lack of secure resection margins, further treatment was decided with adjuvant radiotherapy directed to the anterior mediastinum, at a dose of 54 Gy in 27 fractions. The patient had no immediate nor short-term treatment-related complications, and remained asymptomatic. 

## Discussion

Several studies discuss the potential link between thymoma and other malignancies, both before and after a thymoma diagnosis. Thymomas can present as an incidental finding in asymptomatic individuals in 30% of cases, such as this one. Other than the thymoma, the patient had no other identifiable risk factors for the development of a tumor [2,4]. They can also present with symptoms consistent with an anterior mediastinal mass (30%) or with myasthenia gravis (40%). There is some evidence of a link between Epstein-Barr virus (EBV) and thymic disease [2]. Red cell aplasia and hypogammaglobulinemia are present in 5% of cases of thymomas, in contrast with thymic carcinomas [5]. The three pillars to approach a mediastinal mass are imaging, histopathology, and multidisciplinary expertise. The investigation must rule out an obvious metastatic disease. Surprisingly, the PET scan is not routinely recommended, being optional in cases with aggressive histology or advanced stage to better characterize suspicious lesions [5,6]. The management of these patients should be discussed with a multidisciplinary tumor board team that includes cardiothoracic surgeons to assess resectability and discuss the benefits, and risks, of performing a biopsy as the first approach. These decisions can be complex and depend on the surgeon's experience. The surgical approach with postoperative radiotherapy has a recurrence rate similar to that of the surgical approach alone. The recurrences occur more frequently not in the mediastinal tumor bed but in the intrathoracic pleura, and 30% relapse or present in advance stages. First-line chemotherapy regimes prescribed are cisplatin-anthracycline or cisplatin-etoposide, both in thymoma or thymic carcinoma [5-7].

## Conclusions

We report an extremely rare case of combined thymoma and thymic carcinoma, both rare mediastinal neoplasms. There appears to exist an inherent risk of developing additional neoplasms in patients with thymomas, and when they are asymptomatic, the diagnostic challenge is even greater. This case also aims to emphasize the importance of the three pillars when approaching a mediastinal mass: imaging, histopathology, and multidisciplinary expertise. Depending on the diagnosis, the recommended treatments and follow-up strategies may differ significantly.
